# Shear bond strength of three different metal bracket base designs on human premolars: An *in vitro* comparative study

**DOI:** 10.4317/jced.61166

**Published:** 2024-01-01

**Authors:** Nataly Corahua-Raymi, Seber Guardia-Huamani, Luis Cervantes-Ganoza, Leonor Castro-Ramírez, Carlos López-Gurreonero, Alberto Cornejo-Pinto, César Cayo-Rojas

**Affiliations:** 1 Universidad Nacional Federico Villarreal, Faculty of Dentistry, Lima, Peru; 2 Universidad Inca Garcilaso de la Vega, Faculty of Stomatology, Lima, Peru; 3 Universidad Privada San Juan Bautista, School of Stomatology, Lima, Peru

## Abstract

**Background:**

Adequate bracket-enamel bonding is critical to prevent detachment during orthodontic treatment and minimize any potential delay in results. The aim was to compare the shear bond strength of three metal bracket base designs: laser-structured base, mesh base, and retention grooves base.

**Material and Methods:**

In this experimental in vitro study, 54 human premolars were immersed for one week in 0.1% thymol solution, then placed in distilled water with weekly replacement until the start of the study. The premolars were cemented with brackets of varying base designs: A. Discovery® Smart (laser structured), B. Mini Master® Series (base with mesh), and C. Roth Max (base with retention grooves). All brackets were cemented using TransbondTM XT. A universal testing machine was used to evaluate the shear bond strength at a crosshead speed of 0.75 mm/min. Welch’s one-factor ANOVA with robust variance and Tukey’s post hoc test were used to compare means, with a significance level of *p*<0.05.

**Results:**

The average shear bond strength values were for the bracket with laser-structured base (14.78 ± 5.79 MPa), the bracket with mesh base (9.64 MPa ± 2.54 MPa) and the bracket with retention groove base (15.38 MPa ± 2.67 MPa). It was found that brackets with mesh bases had significantly lower shear bond strength than brackets with laser-structured bases (*p*=0.001) and brackets with retention grooves bases (*p*<0.001). No significant differences were observed between the latter two types of brackets (*p* = 0.893).

**Conclusions:**

The bracket base design influenced in vitro shear bond strength with significantly higher values observed for Roth Max and Discovery® Smart brackets compared to Mini Master® Series brackets.

** Key words:**Shear strength, laser-structured bracket, bracket with mesh base, bracket with retention groove base.

## Introduction

Orthodontic brackets currently serve a critical function in orthodontics by transferring forces from the archwire and ligatures to the periodontal tissues, stimulating tooth movement (1).

However, the failure rate of brackets is varied as it has been reported that between 2% and 20% fail prematurely during treatment (2). A strong fixation of the bracket to the enamel surface is necessary to avoid loss during orthodontic treatment and ensure optimal results within the planned period of time. It is also important to consider that this bond should allow for removal of the bracket at the end of treatment without damaging the enamel (2-4). Reynolds (5) reported an ideal strength range of 5.9 to 7.9 MPa. Diedrich (6) suggests a range from 5 to 10 MPa, while Agarwal *et al*. (4) and Morales *et al*. (7) propose a range of 6 to 8 MPa. It has also been found that values above 20 MPa to 40 MPa may increase the risk of enamel breakage due to exceeding the cohesive forces of the adamantine structure (8,9).

Several factors can affect the adhesion of orthodontic brackets, such as enamel surface nature, conditioning procedure, adhesive type, polymerization type, and bracket base design (3,10,11).

Adhesion at the interface relies on mechanical retention. Therefore, the macroscopic retentive design of the bracket base is of critical importance (2). A high rate of bracket loss is considered detrimental in clinical practice because it increases treatment time and the number of unscheduled appointments (2,3,12).

The different metal bracket bases can be classified into two main groups. In the first group are welded metal bases that can be perforated, with mesh or photo-etched. The second group consists of integral bases that are a single piece with retention grooves, mesh bases, waffle bases, or laser-structured bases (11). When selecting a bracket system, the adhesive strength should be evaluated, as the different characteristics of the bases may affect the effectiveness of the mechanical interlock with the adhesive (13,14).

The study aimed to compare the shear bond strength of three metal bracket base designs (laser-structured base, mesh base, and retention grooves base) on human premolars *in vitro*. The null hypothesis assumes no significant difference in shear bond strength when comparing metal brackets with a laser-structured base, with mesh base, and with retention grooves base.

## Material and Methods

-Study design

This *in vitro* experimental study was conducted at the Universidad Nacional Federico Villarreal (UNFV) and High Technology Laboratory Certificate (ISO/IEC Standard: 17025) in Lima, Peru, during September to October 2021 under approval letter No. 032-2021-COVID-FO-UNFV. This study also followed the CRIS (Checklist for Reporting *In-vitro* Studies) guidelines (15).

-Sample calculation and selection

**Figure 1:**
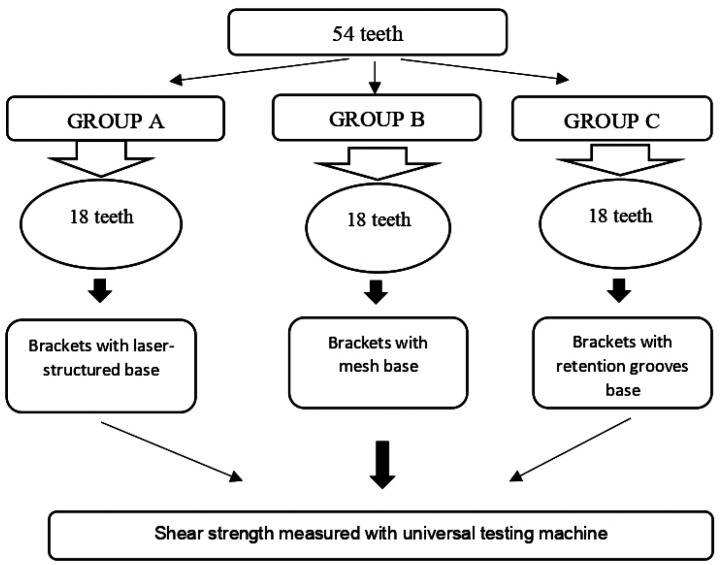
Random distribution of groups according to sample size.

**Figure 2:**
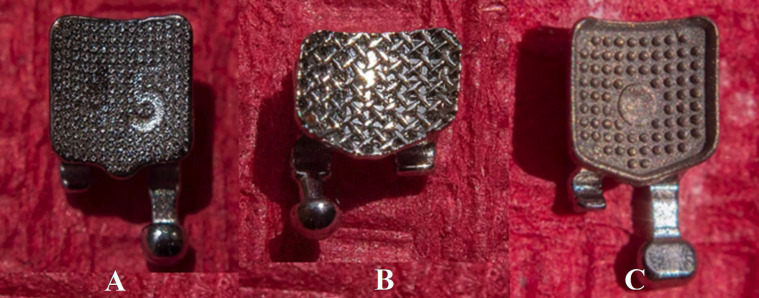
A: laser-structured base, B: mesh base, and C: base with retaining grooves.

Fifty-four healthy human premolars were selected for the experiment. They had been extracted for orthodontic reasons within the previous three months (16,17). Each group had a sample size of 18 teeth (n = 18), calculated using the ANOVA test formula in the statistical software G*Power 3.1.9.7, with α = 0.05 as the significance level, 1 - β = 0.80 as statistical power, and an effect size of 0.849. These data were obtained from a prior pilot study with 12 participants per group. The groups (A, B, and C) were formed using a simple random sampling method without replacement (Figs. 1,2).

-Group A: Brackets with laser-structured base: Discovery® Smart (Dentaurum, Ispringen, Germany), whose base area was 10.18 mm2

-Group B: Brackets with mesh base: Mini Master® Series (American Orthodontics, Sheboygan, NY, USA), whose base area was 10.01 mm2

-Group C: Brackets with retention grooves base: Roth Max (Morelli®, Sorocaba, SP, Brazil), whose base area was 11.32 mm2

-Sample preparation

The extracted teeth underwent initial cleansing with water to remove soft tissues and debris. To prevent bacterial growth and dehydration, they were then immersed for a week in a 0.1% thymol solution (10,14). After this, they were kept in distilled water until the start of the study. The water was changed every week, and the storage did not exceed six months, following ISO/TS11405:2015 regulations (18).

-Mounting of teeth and group formation 

A heavy silicone condensation mold (Zhermack, Badia Polesine, Italy) with an internal diameter and length of 30 mm was used. Vel-Mix™ type IV dental plaster (Kerr Corporation, Orange, CA, USA), Fujirock® EP (GC America Inc, Alsip, Illinois, USA), and Elite Stone (Zhermack, Badia Polesine, Italy) were poured into the mold. The teeth were placed vertically in the mixture with their roots submerged, the longitudinal axis of the tooth was kept parallel to the longitudinal axis of the mould (10). Afterwards, the samples were categorized based on the color of the plaster used: Group A (pink), Group B (blue), and Group C (white). Subsequently, pumice prophylaxis (10,11,13) was carried out utilizing the low speed Micromotor EX-203C (NSK, Tokyo, Japan). It was then subjected to a 10-second jet wash via the triple syringe and subsequently dried (11).

-Study group cementing protocol 

Condac 37 (37 % phosphoric acid etching gel, FGM, Joinville, Santa. Catarina, Brazil) was applied for 15 seconds on the vestibular surface of the clinical crown. The surface was then washed and dried with air. A thin layer of Transbond™ XT Primer (3M™ Unitek, Monrovia, CA, USA) was applied on the enamel by rubbing it for 10 seconds. Subsequently, with the assistance of a bracket holder forceps, a small amount of Transbond XT resin was applied over the bracket base and positioned at the center of the clinical crown. The correct bracket positioning was verified with a bracket positioner (Morelli®, Sorocaba, SP, Brazil) followed by resin excess removal with a dental explorer. Light curing of the mesial and distal sides of the bracket (2,10,11) was then performed using a Valo Cordless® LED (Light-Emitting Diode) unit (Ultradent© in South Jordan, UT, USA) at an intensity of 1000 mW/cm². Each bracket side was light cured for 10 seconds.

-Storage and shear bond strength

**Figure 3:**
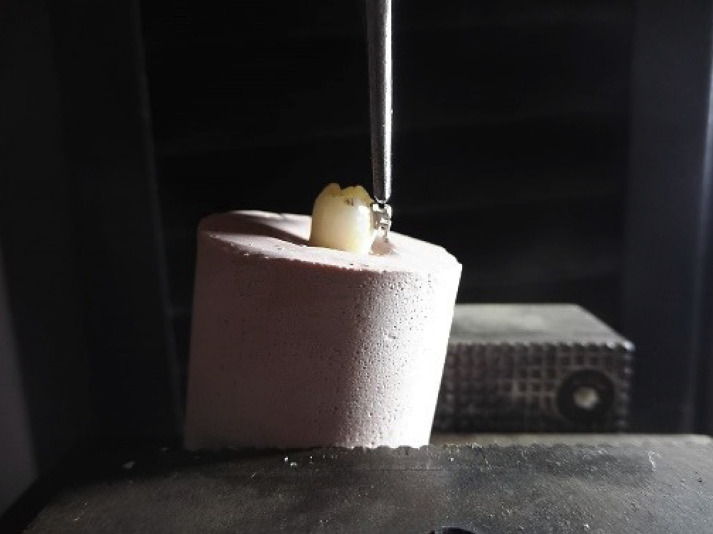
Shear bond strength of metal bracket.

The specimens were immersed in distilled water at 37°C for 24 hours (3,10,19). A universal testing machine (CMT-5L, 7419, LG, Seoul, Korea) with a crosshead speed of 0.75 mm/min was used to perform the shear bond strength test. The force was applied to the brackets through a blade in the occlusogingival direction at the enamel/bracket interface until detachment occurred (4) (Fig. 3). The computer linked to the testing machine provided the results. The obtained results in Newtons were registered onto the data collection card and subsequently converted to MPa through simple division of the force by the bracket’s area.

-Statistical analysis

The collected data were stored in Microsoft® Excel 2019 and exported to analyze using SPSS V.28.0 statistical program. Descriptive analysis utilized the mean, standard deviation, and maximum and minimum values of the three study groups. The inferential analysis involved testing normal distribution and homoscedasticity using the Shapiro Wilk and Levene tests. We employed Welch’s parametric one-factor intergroup ANOVA test with robust variance and used Tukey’s post-hoc test for multiple comparisons of means between the study groups. All statistical analyses were conducted at a significance level of *p*<0.05.

## Results

**Figure 4:**
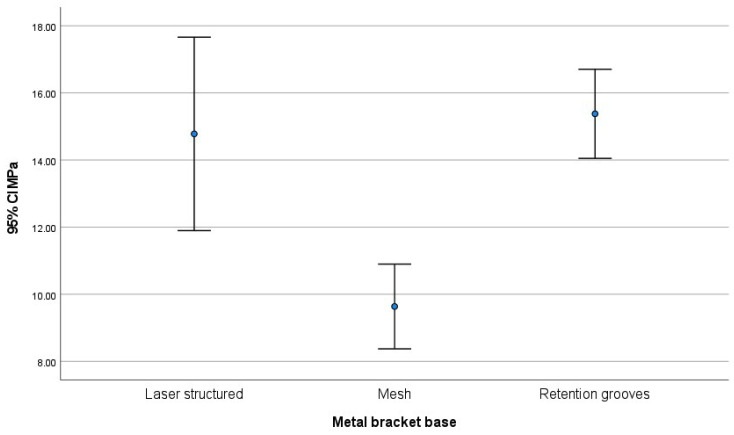
Average shear bond strength (MPa) of metal bracket bases with 95% confidence intervals.

The brackets’ average shear bond strength values (measured in MPa) were as follows: with laser structured base had a value of 14.8 ± 5.8 MPa, with mesh base had 9.6 ± 2.5 MPa, and retention grooves base had 15.4 ± 2.7 MPa. The study showed that brackets with mesh base had significantly lower shear strength (MPa) than brackets with laser structured base (*p* = 0.001) and brackets with retention grooves base (*p*<0.001). However, there were no significant differences observed between the latter two types of brackets (*p* = 0.893) (Table 1, Fig. 4).

**Table 1:**
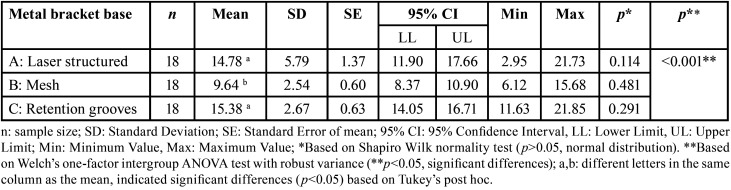
Descriptive values of shear bond strength (MPa) and comparison of means of the study groups according to the base of the metal brackets used.

## Discussion

The study aimed to compare the shear bond strength of three metal bracket base designs: laser structured base, mesh base, and retention grooves base. Results showed that brackets with mesh base had significantly lower shear bond strength than those with retention grooves base and laser structured base, leading to the rejection of the null hypothesis.

The different base designs yielded different results in terms of shear strength, which is consistent with previous studies conducted by Cozza *et al*. (11), Sorel *et al*. (20), and Castillo *et al*. (21). However, the mean values of these studies were generally lower when compared to the results of the present study. Differences in methodology may be the reason for this discrepancy. Some authors chose to use a self-curing adhesive such as No-mix (Dentaurum, Ispringen, Germany), while others used Transbond XT light-cured at 380 mW/cm2 with crosshead speed of 1 mm/min (11,20,21).

Chaudhary *et al*. (22), Molina *et al*. (23), and Lo Giudese *et al*. (24) found no significant differences between their study groups, which contradicts the results of the present study. This disparity could be attributed to the use of brackets with rail or Treadlock base, double-mesh base, and horizontal groove base, along with a different highly transparent adhesive (Heliosit, Ivoclar Vivadent srl, Italy). Therefore, it can be stated that there are several factors that influence the adhesive bonding values of metal brackets (3,10,11).

Among the bracket systems analyzed in the study, Roth Max (retention grooves base) exhibited one of the highest average shear strengths. This is likely because of the factory micro-sandblasting, which may enhance tooth enamel retention, as well as the presence of micro-pins that increase bonding surface area and anchorage to the adhesive (25). Kilponen (2) and Hodecker (3) previously noted that sandblasting, silanization of the bracket base, and chemical conditioning improve the bond strength of metal brackets. Discovery® Smart, another type of bracket with high average shear strength, has a laser-structured base that undergoes treatment with a powerful Nd:YAG laser. This laser melts and evaporates the metal, forming hole retentions that likely provide a combination of macro and micro retention, thus offering better bond strength (4,19). The Mini Master® series exhibited decreased shear strength. This bracket features an 80-gauge welded mesh on an etched foil base and undergoes photochemical etching, resulting in the formation of porosities that provide retention (19). It is possible that the lower values obtained from this bracket are attributed to the higher retentiveness of integral bases, such as Roth Max and Discovery® Smart, compared to metal mesh bases (22,26). The presence of solder in the base of the Mini Master® series may result in air entrapment, leading to voids under the solder points. As a consequence, air retention decreases, and the adhesive may not penetrate adequately, exposing the area to marginal leakage and potential joint failure (1,26).

The results of the present study show that the Roth Max, Discovery® Smart, and Mini Master® were all within accepted clinical values, with mean measurements of 15.38 MPa, 14.78 MPa, and 9.64 MPa, respectively. It is important to note that none of these values exceeded 20 MPa at 40 MPa, which is a critical threshold, as previous studies (8,9) have reported that exceeding this limit may indicate an increased risk of enamel damage during removal procedures. However, research indicates that *in vivo* bonding strength is lower than *in vitro* bonding strength (3,27). Pickett *et al*. used an intraoral debonding device to compare *in vitro* and *in vivo* results and found that the bond strengths *in vivo* (5.47 MPa) were significantly lower than those recorded *in vitro* (12.82 MPa) (27). The decrease in bond strength can be attributed to the brackets’ exposure to the oral environment, including acid, saliva, and masticatory forces over time.

On the other hand, the area of the bracket bases used were Roth Max (11.32 mm2) and Discovery® Smart (10.18 mm2), Mini Master® Series (10.01 mm2) respectively. Izquierdo *et al*. (13) and Wang *et al*. (28) reported that larger size bases produced higher bond strength than smaller size bases, which is in agreement with our results, as the mean shear bond strength values were Roth Max (15.38 MPa), Discovery® Smart (14.78 MPa) and Mini Master® Series (9.64 MPa).

The importance of this study lies in the quantitative determination of the metal bracket base design with the highest shear resistance, which enables the clinician to select the optimal bracket base for reducing the likelihood of loosening. As a result, this avoids hindering the treatment mechanics and decreases treatment duration, ultimately preventing needless costs for the patient (2,3,12).

A methodological strength of the present study was verifying the proper adaptation of the samples to the universal testing machine. One operator conducted all procedures to ensure consistent pressure during bracket positioning, uniform adhesive thickness, and careful removal of excess without bracket displacement. Furthermore, we utilized the Transbond™ XT adhesive system (primer and resin), which is widely recognized as the conventional bonding system (13). Additionally, all groups underwent the same light curing protocol to ensure that any discrepancies in strength values could be ascribed to differences in bracket base design. Transbond™ XT provides an extended period of workability, enabling adjustments to the final bracket position. The formula features methacrylate phosphoric acid esters, stabilizers, and photosensitizers, which facilitate quick attainment of optimal physical properties, allowing prompt treatment following adhesive application. However, due to the low amount of carboxyl groups in the monomers, there is no chemical bonding with the enamel. As a result, the enamel must be conditioned beforehand and a dry working field is necessary (3,7,20).

As an *in vitro* study, caution must be exercised when extrapolating results to the oral environment, where moisture contamination significantly reduces adhesion (11). Furthermore, variables such as patient age and gender, type of malocclusion, appliance care, overall diet, and trauma are important factors that influence bond failure (22,26).

Future research should consider the size of the bracket base, given that larger bases reportedly produce greater bond strength than smaller ones. It may be beneficial to use thermocycling to simulate conditions in the oral environment when evaluating adhesive strength after one year of clinical aging (2,11,22,28). It would be beneficial to assess the nickel concentrations within the brackets studied here, as it has been reported that the insertion of fixed orthodontic appliances can cause hypersensitivity reactions due to a significant increase in the level of nickel in saliva (29).

Furthermore, it is recommended that future studies examine the adhesive remnant index and post-descementation enamel damage to determine the maximum safe limit in MPa for preventing enamel damage. Various authors have reported different maximum limits, ranging from 20 MPa to 40 MPa (8,9) to 13 MPa to 14 MPa (30), indicating a lack of consensus.

## Conclusions

Recognizing the limitations of this *in vitro* study, it can be concluded that the bracket base had an impact on shear bond strength, with the retention grooves base and laser-structured base exhibiting significantly higher values than the mesh base. It is important to note that these values may be considerably lower in an *in vivo* environment. Thus, clinicians are advised to consider utilizing metal brackets with integral bases alongside standard adhesives for enhanced treatment efficiency.
